# Silver-containing composites based on copolymers of β-cyclodextrin and TiO_2_ for enhanced photocatalytic degradation of methyl orange in environmental water†

**DOI:** 10.1039/d4ra08901d

**Published:** 2025-05-30

**Authors:** Serhii Kobylinskyi, Sergii Sinelnikov, Larysa Kobrina, Yuliia Bardadym, Sergii Riabov

**Affiliations:** a Institute of Macromolecular Chemistry, National Academy of Sciences of Ukraine 48, Kharkivske shose Kyiv 02155 Ukraine sergii.riabov@gmail.com

## Abstract

This study aims to develop effective photocatalysts by combining the photocatalytic properties of TiO_2_ and Ag nanoparticles (AgNPs) with the adsorption ability of β-cyclodextrin-containing polymers (CDPs). For this purpose, TiO_2_/CDP/AgNP hybrid catalysts were prepared *via* radical and thermal polymerization of cyclodextrin derivatives with trimethylolpropane trimethacrylate on the TiO_2_ surface, followed by introduction of 1 wt% silver nanoparticles into their bulk *via* reduction of Ag^+^ salts under UV irradiation or using NaOH. The structure of the nanocomposite photocatalysts was investigated through X-ray diffraction (XRD), Fourier transform infrared spectroscopy (FTIR), and thermal gravimetric analysis (TGA), and particle size distribution was determined *via* dynamic light scattering (DLS). The photocatalytic degradation process was monitored *via* UV-vis spectroscopy in an aqueous environment at pH 2.5. The best photodegradation results for methyl orange (MO) were obtained using samples prepared from β-CD methacrylate and silver methacrylate. In this case, complete decolorization of the MO solution was achieved within 10 min at pH 2.5, which is 7.5 times faster than that achieved by pure TiO_2_ and 4 times faster than that that achieved by the TiO_2_/CDP sample.

## Abstract 2

Schematic route for the modification of a TiO_2_ surface by copolymers and AgNPs.

## Graphical abstract

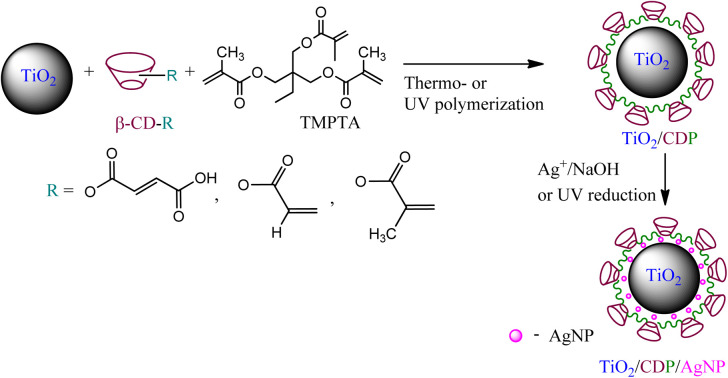

## 1. Introduction

Water pollution caused by organic compounds, such as dyes, is a serious environmental problem that threatens ecosystems and human health. Dyes are widely used in the textile, food, paper and cosmetic industries. Owing to their high resistance to biodegradation, they persist in the environment for long periods, which make them particularly hazardous to water systems.^1^ Traditional water treatment methods, such as physical (activated carbon adsorption and filtration), chemical (coagulation and chlorination) and biological (biodegradation) processes, have a number of limitations in removing dyes, especially those that are stable and toxic even at low concentrations.^2^

Photocatalytic degradation using titanium dioxide (TiO_2_)-based nanomaterials is one of the most promising methods for water purification through the removal of persistent organic pollutants, including dyes. TiO_2_ has attracted considerable attention owing to its high activity, chemical stability, and environmental safety.^3–6^ However, there are a number of drawbacks that limit the widespread industrial application of this technology. One of the major disadvantages of TiO_2_ is that it is only activated by ultraviolet radiation, which comprises only 5% of the solar spectrum. This severely limits the use of solar energy for photocatalysis under natural conditions. Even in the presence of UV light, the efficiency of photocatalytic reactions is often reduced owing to the rapid recombination of excited electrons and holes. This shortens their lifetime and likelihood of interaction with organic contaminant molecules. For effective degradation, pollutants must be adsorbed onto the catalyst surface. However, many dyes exhibit low affinity for the TiO_2_ surface, reducing their contact and, consequently, reaction efficiency. The photocatalytic degradation of dyes can produce toxic intermediates that pose a threat to aquatic organisms and humans. This requires further study of the kinetics and mechanisms of degradation to prevent the formation of hazardous by-products. In addition to process efficiency, it is important to ensure the stability and long-term performance of photocatalytic materials in aqueous environments because some materials may lose activity owing to particle aggregation or chemical modification during the reaction.

This study aims to modify TiO_2_ to solve these problems. One of the directions is the doping of TiO_2_ with metals (Ag, Pt, Au) and non-metals (nitrogen, carbon, fluorine) to extend its activity in the visible light range.^6^ Another promising area is the creation of hybrid nanocomposites based on TiO_2_ with organic molecules such as cyclodextrins (CDs), which can improve the adsorption of organic compounds on the catalyst surface and reduce electron and hole recombination. These solutions significantly improve the efficiency of processes and make them suitable for practical applications.^7–9^

Several reports have described the preparation of TiO_2_/β-CD and TiO_2_/β-CD/Ag composites.^10–23^ García-Díaz *et al.*^10^ studied the use of TiO_2_ microspheres coated with β-CD polymer obtained from condensation polymerization with tetrafluoroterephthalonitrile, resulting in the formation of a cross-linked, covalently bonded β-CD film. The use of β-CD as a coating improves the stability of the microspheres and increases the duration of the catalytic effect to at least 1000 hours, which was studied in the photocatalytic degradation of bisphenol A (BPA) in wastewater. Cyclodextrin improves BPA adsorption, accelerating its degradation. Coated TiO_2_ microspheres have a more stable surface and better charge separation, which reduces the recombination rate. Sangari *et al.*^11^ investigated the effect of β-cyclodextrin on the photocatalytic degradation of Metanil Yellow dye using TiO_2_. β-CD was utilized to enhance the photocatalytic activity by increasing the adsorption of the dye on the TiO_2_ surface. β-CD forms inclusion complexes with dye molecules, which accelerates their transfer to the photocatalyst surface and improves the reaction under UV light. The study showed a significant increase in the degradation rate of methylene yellow compared to the use of TiO_2_ alone. In,^7^ a β-CD polymer/TiO_2_ composite was prepared by the *in situ* growth of TiO_2_ on a β-CDP matrix synthesized by epichlorohydrin crosslinking. The photodegradation efficiency of this composite toward tetracycline was attributed to its mesoporous structure, large pore volume and high surface area, which provided many active sites for photocatalytic activity and effectively improved the separation of photogenerated electrons and holes. Colpani *et al.*^12^ considered the functionalization of TiO_2_ with carboxymethyl-β-cyclodextrin and lanthanum doping. Such modifications improve the absorption of visible light and increase the activity of photocatalysts for the degradation of organic pollutants. This study^13^ investigates a floating photocatalytic membrane based on TiO_2_ doped with silver and β-cyclodextrin (β-CD). These membranes were designed to improve their adsorption and photocatalytic activity under visible light. Silver broadens the absorption spectrum of visible light, while β-CD enhances the adsorption of organic pollutants. The synergy between Ag and β-CD promotes more efficient photocatalytic decomposition. The membranes showed high efficiency in the dynamic adsorption of pollutants and higher activity under visible light compared to pure TiO_2_. In,^14^ a hybrid system based on TiO_2_ decorated with gold (Au) nanoclusters and cyclodextrin was investigated. In the developed hybrid material, the combination of the properties of SH-β-CDs, Au NCs and TiO_2_ NPs led to a significant improvement in the photodegradation of methyl orange (∼98%, *t* = 10 min) due to the increased availability of catalytic centers and inhibition of electron-hole pair recombination. In the article,^15^ titanium dioxide nanoparticles doped with Ag and β-cyclodextrin (β-CD) were synthesized on activated carbon (AC). The resulting Ag-β-CD/TiO_2_/AC composite exhibited high photocatalytic activity (98.4%) and rate constant for naphthalene under visible light irradiation. In our previous articles, we studied the effect of both the original cyclodextrin and its derivatives,^19,20^ as well as the cross-linked copolymers of cyclodextrin methacrylate and maleate as additives^21^ that are grafted onto the surface of titanium dioxide through silicon derivatives,^22,23^ on the photodegradation of methyl orange. It has been shown that β-CD maleates, both alone and as part of cross-linked copolymers, have a positive effect on the photodegradation rate of methyl orange in distilled water.

At the same time, there is a lack of data in the literature on the effect of modification of TiO_2_ by cross-linked polymers based on CD together with AgNPs on its photocatalytic activity, and the effect of CD, the chemical composition of the silver salt used, and the method of silver ion reduction on the formation of AgNPs has not been studied, but such information is important from scientific and practical points of view. Thus, in this study, we elaborated polymer composites based on TiO_2_, cyclodextrin derivatives: methacrylate, maleate and AgNPs. The photocatalytic activity of the obtained composite catalysts towards methyl orange as a model object was investigated in environmental water media.

## 2. Experimental part

### 2.1. Reagents

β-Cyclodextrin (β-CD) (Cavamax W7, Wacker), titanium dioxide (TiO_2_), silver nitrate (AgNO_3_), silver nanoparticles (AgNPs) (<100 nm), methacrylic anhydride (MethA), maleic anhydride (MA), acryloyl chloride (AC), trimethylolpropane trimethacrylate (TMPTA), α,α′-azoisobutyronitrile (AIBN), α,α-dimethoxy-α-phenylacetophenone (ketal), trimethylamine, trisodium citrate, ascorbic acid, acetone, dimethylformamide (DMF), butyl acetate (BA), hydrochloric acid (37%), sodium hydroxide were supplied by Sigma-Aldrich (Germany). A stock solution of 1.5 g L^−1^ methyl orange was prepared by dissolving 0.75 g of MO in 500 ml of double-distilled water.

### 2.2. Synthesis of silver(i) precursors

Silver citrate (Ag_3_Citr) was synthesized by precipitation from the reaction between AgNO_3_ and sodium citrate at a molar ratio of 3 : 1.^24^ This reaction resulted in the formation of a white precipitate, which was filtered, washed with distilled water, and dried at 50 °C away from light.

### 2.3. Instrumental methods

1H NMR spectra were acquired on a Bruker DPX 300 NMR spectrometer (Germany) using DMSO-d6 as solvent. UV-vis spectra were recorded using a UV-vis spectrophotometer UV-2401 PC (Shimadzu, Japan) in the frequency range 190–800 nm. FTIR spectra were obtained using a Tensor-37 Fourier Transform Infrared Spectrometer (Bruker, Germany) in the range 400–4000 cm^−1^. Thermogravimetric analysis of the samples was performed using a TGA Q50 (TA Instruments, USA) in an air atmosphere in the range of 20–550 °C at a heating rate of 20 °C min^−1^ and using a Mettler Toledo simultaneous TGA/SDTA851 thermogravimetric analyzer in a N_2_ atmosphere at a gas flow rate of 100 ml min^−1^ at a heating rate of 10 °C min^−1^ in the range of 20–1000 °C. X-ray diffraction (XRD) of the photocatalysts was recorded by a PAN Analytical X'Pert Pro high-resolution diffractometer using CuK_α_ radiation (*λ* = 1.54 Å). XRD profiles were fitted with the Voigt functions. The instrumental broadening correction was performed using a polycrystalline SiO_2_ standard and the Voigt function method. The average crystallite size (*D*) and interplanar spacing (*d*) of TiO_2_ and the prepared composites were calculated using the Scherer and Bragg equations, respectively:
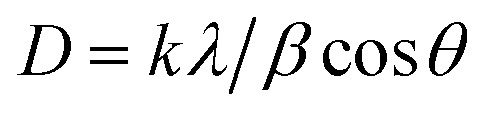

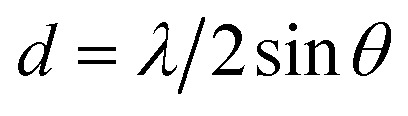
where *K* is a constant depending on the particle morphology of the scattering object and varies from 0.89 to 1.39 (*i.e.* for particles of unknown shape – 0.9, for spherical particles – 0.94); *β* is the full width at half maximum (FWHM) of the peak; *θ* is the Bragg angle; *λ* is the wavelength of characteristic X-ray radiation (*λ* = 1.54 Å for CuKα radiation).

The lattice parameters of solids can be calculated using the following formula:
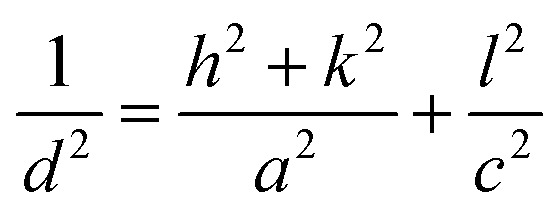
where *d* is the interplanar spacing; *h*, *k* and *l* are the Miller indices; *a* and *c* are the lattice parameters;

The particle size of each sample was measured in triplicate at 25 °C by dynamic light scattering using a Litesizer 500 (Anton Paar, Austria) (40 mW red semiconductor laser of 658 nm) at a scattering angle of 175°. The TiO_2_ samples were ultrasonically dispersed in water at a concentration of 1 mg ml^−1^ for 30 min before testing. The size measurements of each sample (∼1 ml per sample) were presented in terms of three metrics, intensity-based size, number-based size and volume-based size. The zeta potential was determined using the Smoluchowski approximation from the electrophoretic mobility measurements based on Henry's equation. The measuring angle was automatically set to 15° by the instrument, and the measuring range was −600 to +600 mV.

For the quantitative determination of silver, the sample (0.1000 g) was suspended in 10 ml of dilute HNO_3_ (1 : 1) and CO_2_-free distilled water (total 50 ml) and analyzed in duplicate by atomic absorption spectroscopy (spectrometer C-115M1) at 328.1 nm.

The N_2_ adsorption/desorption isotherms were performed at −196 °C on a Quantachrome instrument (NOVA-2200 Gas Sorption Analyzer, USA) using the Nova Win 2.0 software. Before the measurements, the samples were outgassed at 150 °C for 20 h. The specific surface area of the materials was calculated using the Brunauer–Emmet–Teller (BET), the Barrett–Joyner–Halenda (BJH) and Dollimore–Heal (DH) methods. The pore size distribution of the samples was calculated using the quenched solid density functional theory (QSDFT) method with a slit/cylindrical pore model. The total pore volume (*V*_total_) was calculated from the volume of adsorbed nitrogen converted to liquid at a pressure close to *P*/*P*_0_ = 1. The BJH and DH methods were used to calculate the mesopore volume (*V*_meso_). The average pore radius (*R*_av_) was determined from the total pore volume (*V*_total_) and its specific surface area (*S*_BET_) using the equation *R*_av_ = 2*V*_total_/*S*_BET_. The micropore volume (*V*_micro_) was calculated from the difference between *V*_total_ and *V*_meso_ using the Dubinin–Radushkevich (DR) and Saito–Foley (SF) methods.

### 2.4. Procedures for the reduction of silver ions in water solution

Thus, the reduction of Ag^+^ ions was fulfilled with NaOH in the presence of β-cyclodextrin in aqueous solution as follows: 0.4 ml of β-cyclodextrin solution (0.7 μmol) with a concentration of 2 g L^−1^, 0.2 ml of AgNO_3_ (6.31 μmol), and 28.9 ml of distilled water were placed in a glass tube and stirred for 2 min at room temperature and then 0.5 ml of NaOH (0.0105 mmol) was added. The molar ratio of β-CD to AgNO_3_ and NaOH was 1 : 9 : 15. This solution was heated at 85 °C for 6 hours. The synthesis of TiO_2_/AgNPs samples was conducted under the same conditions, with the addition of the required amount of titanium dioxide. After reduction, the product was centrifuged at 4000 rpm, washed with distilled water, and dried at 70°C. A similar sample was obtained using silver methacrylate. The silver content determined by atomic absorption spectroscopy was 1 wt%.

### 2.5. Synthesis of β-cyclodextrin derivatives (monomers)

Synthesis of acylated β-cyclodextrin derivatives (β-CD-(Meth)_*n*_ and β-CD-(Mal)_*n*_).^19,21^

#### 2.5.1. Synthesis of β-cyclodextrin methacrylate

To 1.135 g (1 mmol) of β-CD dissolved in 5 ml of DMF, 0.89 g (5.3 mmol) or 1.226 g of methacrylic anhydride (7.3 mmol) previously mixed with 0.5 mmol of trimethylamine (TEA) was added, then stirred for 8 h at 40 °C and left for 24 h at room temperature. The product was precipitated in 40 ml of acetone, and the precipitate was washed several times with acetone. The product was dried at 50 °C until a constant weight was achieved. The degree of substitution (DS) was 5 and 7 based on the NMR data.

#### 2.5.2. Synthesis of β-cyclodextrin maleate

To 1.135 g (1 mmol) of β-CD dissolved in 5 ml of DMF, 0.519 g (5.3 mmol) of maleic anhydride previously mixed with 0.5 mmol of trimethylamine (TEA) was added, then stirred for 4 h at 80 °C and left for 24 h at room temperature. The product was precipitated in 40 ml of acetone, and the precipitate was washed several times with acetone. The product was dried at 50 °C to a constant weight.

#### 2.5.3. Synthesis of β-cyclodextrin acrylate^25^

1.135 g of β-CD (1 mmol) was dissolved in 8 ml of DMF, and 0.633 g (7 mmol) of acryloyl chloride was added. After stirring, 7 mmol of triethylamine was added, stirred for 2 h and left at room temperature for 1 day. The triethylamine hydrochloride precipitate was filtered and discarded, and the product solution was precipitated into 85 ml of acetone, filtered, washed with two portions of acetone each, and dried at 40 °C to a constant weight.

### 2.6. Preparation of cross-linked β-cyclodextrin-containing polymers on TiO_2_ surfaces

The thermo-polymerization of monomers (β-cyclodextrin methacrylate or β-cyclodextrin maleate) on the TiO_2_ surface was carried out in a mixture (30 ml) of i-PrOH/H_2_O (50 : 50 v/v), initiator AIBN, and, in the case of UV polymerization, in H_2_O using ketal as initiator.^26^ Cross-linker in both cases was trimethylolpropane trimethacrylate (TMPTA). The ratios of the starting components are given in Table 1. To perform thermo-polymerization, the mixture composition was stirred on a magnetic stirrer at a temperature of 80 °C for 6 h; in turn, UV polymerization was performed at room temperature for 90 min. The products were then centrifuged, washed several times with distilled water, and dried at 70 °C to constant weight.

**Table 1:** Ratio of components involved in radical polymerization on TiO_2_

Sample	Content of initial components, g	Solvent/initiator	Silver precursor/reduction
**TiO**_**2**_ **/TMPTA/CD(Mal)**_**5**_			
TiO_2_-P1	0.5/0.0505/0.241	i-PrOH/H_2_O/AIBN	—
TiO_2_-P2	0.5/0.087/0.412	i-PrOH/H_2_O	—

**TiO**_**2**_ **/TMPTA/CD(Meth)**_**5**_			
TiO_2_-P3	0.5/0.023/0.101	H_2_O/ketal	—
TiO_2_-P4		H_2_O/AIBN	—
TiO_2_-P5Ag	0.5/0.023/0.101	H_2_O/UV polymerization/ketal	0.0093 g AgNO_3_/UV reduction
TiO_2_-P6Ag			0.0106 g Ag_Meth_/UV reduction
TiO_2_-P7Ag			0.094 g Ag_3Citr_/UV reduction
TiO_2_-P8Ag			0.094 g Ag_3Citr_/reduction by NaOH

**TiO**_**2**_ **/TMPTA/CD(Ac)**_**7**_			
TiO_2_-P9	0.5/0.024/0.108	H_2_O/UV polymerization/ketal	—

**TiO**_**2**_ **/TMPTA/CD(Meth)**_**7**_			
TiO_2_-P10	0.5/0.024/0.108	H_2_O/UV polymerization/ketal	—
TiO_2_-P10Ag	0.2 (TiO_2_-P10)	H_2_O/UV polymerization/ketal	0.0032 g Ag_3Citr_/reduction by NaOH
TiO_2_-P11Ag	0.5/0.024/0.108		0.01 g Ag_3Citr_/reduction by NaOH
TiO_2_-P12Ag			0.01 g Ag_3Citr_/UV reduction
TiO_2_-P13Ag			0.0109 g Ag_Meth_/reduction by NaOH
TiO_2_-P14	0.5/–/0.12	DMF/BuAc	—
TiO_2_-P15Ag	0.5/–/0.12		0.0108 g Ag_Meth_/ascorbic acid/KOH

### 2.7. Preparation of silver-containing composites

Silver-containing composites were obtained by reduction of silver ions of appropriate salts-nitrate, citrate, and methacrylate, at the rate of 1% by weight in the final product (TiO_2_/CDP) using NaOH, ascorbic acid (AA) or UV irradiation. TiO_2_ samples containing the copolymer and silver nanoparticles (AgNPs) were prepared without separation of the intermediate product (copolymer), except for the TiO_2_P10-Ag one. After UV polymerization of CD monomers, an appropriate amount of the corresponding silver salt and the required quantity of NaOH were added. The mixture was then stirred on a magnetic stirrer at a temperature of 80 °C for 6 hours. The product was then centrifuged, washed several times with distilled water, and dried at 70 °C up to constant weight was achieved.

The TiO_2_-P14 and TiO_2_-P15Ag composites were prepared without TMPTA as follows: 0.12 g CD(Meth)_7_ was dissolved in 1 ml DMF, and then 10 ml butyl acetate and 0.5 g TiO_2_ were added. The mixture was refluxed with stirring for 1.5 h, cooled, and then the product was filtered, washed with EtOH, distilled water, and dried at 70 °C. The TiO_2_-P15Ag composite was similarly synthesized. Silver methacrylate was added to the mixture after 30 min of refluxing TiO_2_ with CD(Meth)_7_, after 5 min of stirring solution of ascorbic acid and KOH in EtOH was added in the molar ratio AgMeth : AA : KOH 1 : 0.5 : 0.25.

### 2.8. Photocatalysis experiment

The photodestruction experiments were carried out in a 100 ml quartz test tube, to which the required volume of MO solution and 40 mg of photocatalyst were added. The total volume was 80 ml. The resultant suspension was exposed to UV irradiation under stirring. The irradiation source was a system of three 8 W UV lamps with wavelengths of 365 nm (2 lamps) and 254 nm (1 lamp) placed vertically on three sides of the test tube.^20^ The following reagent concentrations were used in the study: TiO_2_ 0.5 g L^−1^, MO – 25 mg L^−1^, artesian water was acidified to pH 2.5 from pH 7.3–7.5. To study the kinetics of photodegradation, the MO concentration was measured at certain intervals (after centrifugation of the solid phase) and determined spectrophotometrically at 504 nm.

## 3. Results and discussion

### 3.1. Synthesis of silver citrate and methacrylate

**Fig. 1:**
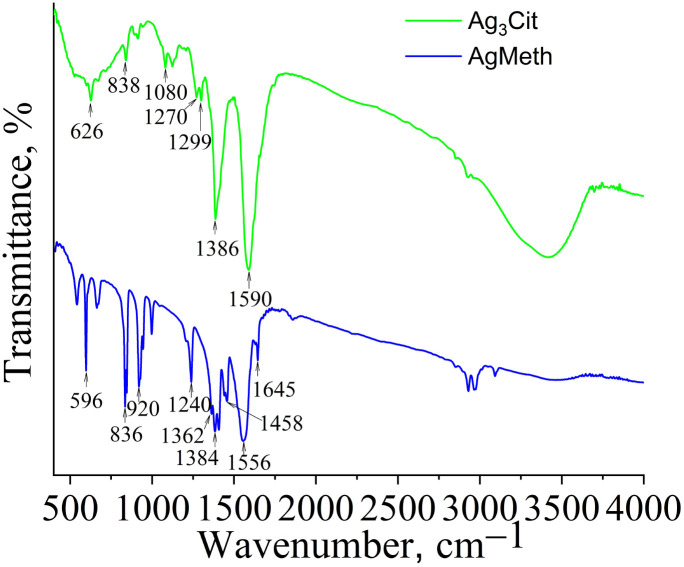
FTIR spectra of silver citrate and methacrylate.

Silver methacrylate (Ag_Meth_) was obtained by mixing aqueous solutions of silver nitrate and sodium methacrylate at an equimolar ratio. The formation of these silver salts was confirmed by FTIR spectroscopy (Fig. 1). In the spectra of silver citrate and methacrylate, the strong bands at 1590, 1556 cm^−1^ and 1386, 1384 cm^−1^ characterize the asymmetric and symmetric stretching vibrations of carboxylate groups coordinated with Ag^+^.

### 3.2. Synthesis and characterization of the TiO_2_/CD/copolymers and TiO_2_/CD/copolymers/Ag composites

#### 3.2.1. Synthesis of composites

**Fig. 2:**
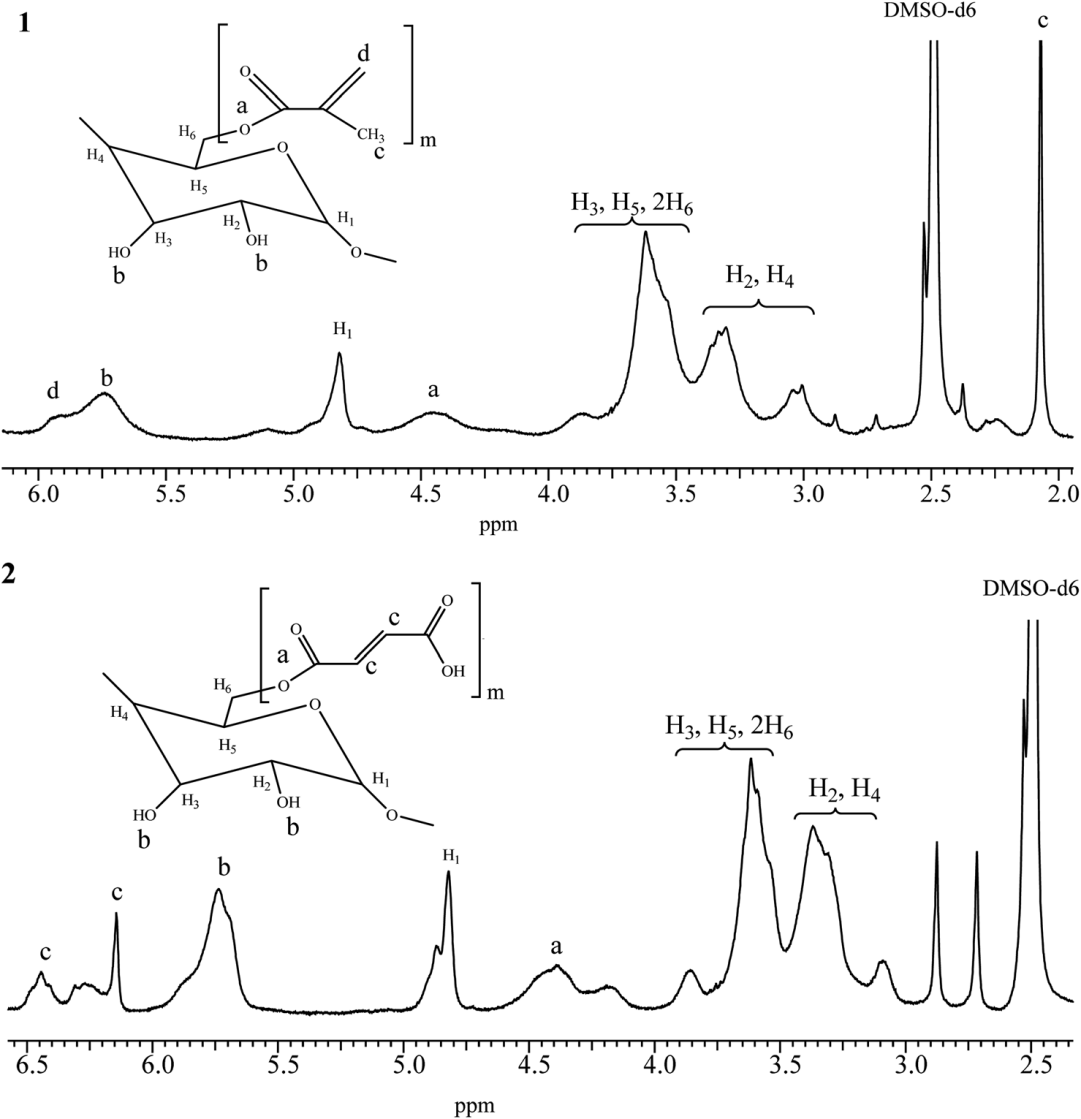
^1^H NMR spectra of β-cyclodextrin derivatives: (1) – β-CD methacrylate (*m* = 5), (2) – β-CD maleate (*m* = 5).

**Fig. 3:**
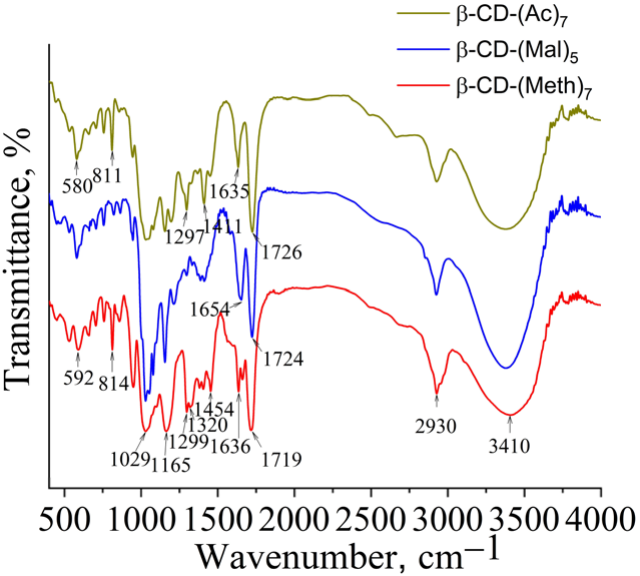
FTIR spectra of β-CD monomers.

Synthesis of β-cyclodextrin derivatives (monomers). The chemical structures of the obtained products were confirmed by ^1^H NMR (Fig. 2) and FTIR (Fig. 3) spectra.

**Scheme 1:**
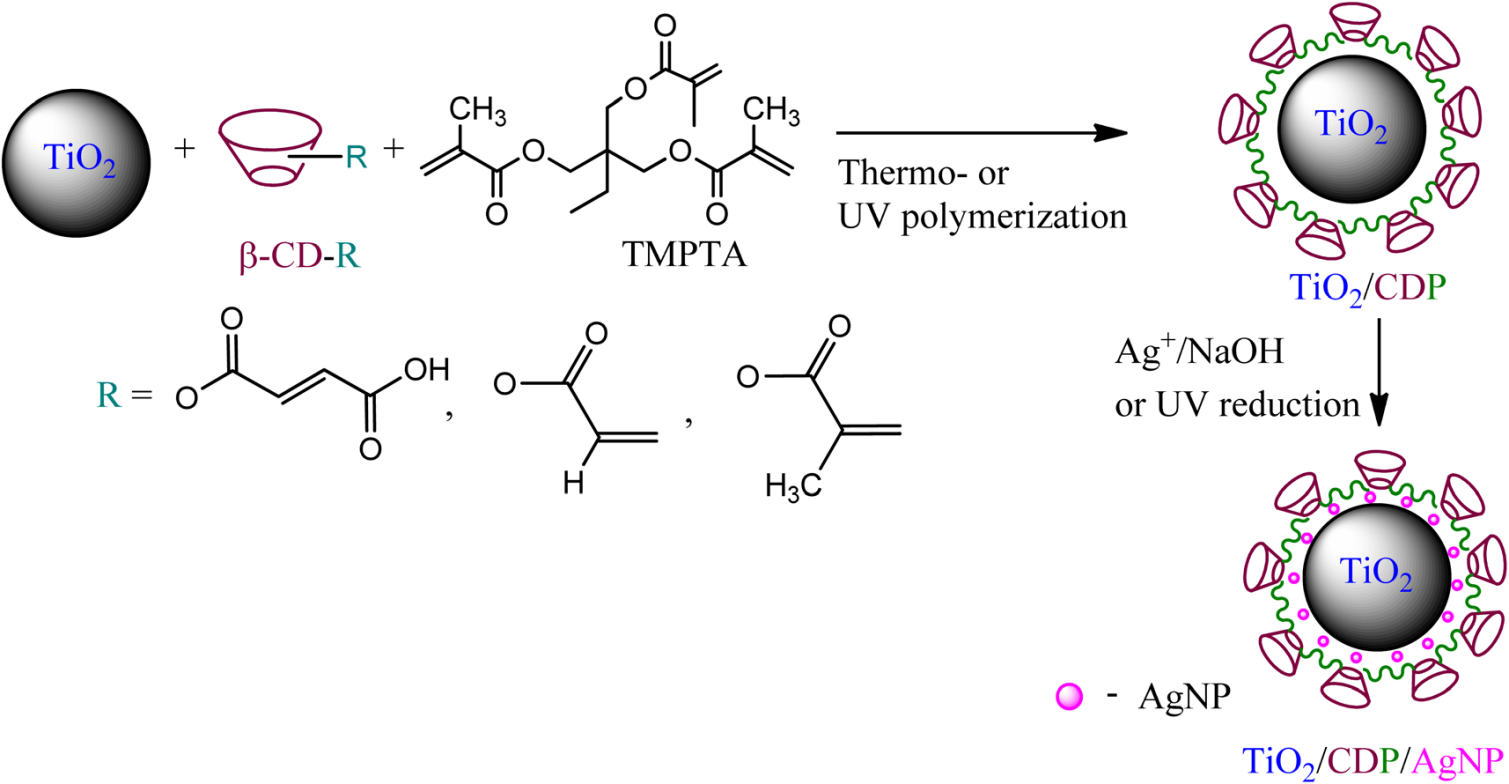
Schematic route for modification of the TiO_2_ surface by copolymers and AgNPs.

First, TiO_2_/CD copolymers' objects were synthesized by thermo- or UV polymerization of β-cyclodextrin monomers (methacrylate or maleate) on the TiO_2_ surface, involving trimethylolpropane trimethacrylate (TMPTA) as a cross-linker at different temperature conditions. In the second stage, composites with silver nanoparticles (1 wt%) (TiO_2_/CDP/AgNPs) were obtained by reduction of silver ions from the appropriate salts (nitrate, citrate, methacrylate) using NaOH, UV irradiation or ascorbic acid Scheme 1.

#### 3.2.2. Characterization of composites

**Fig. 4:**
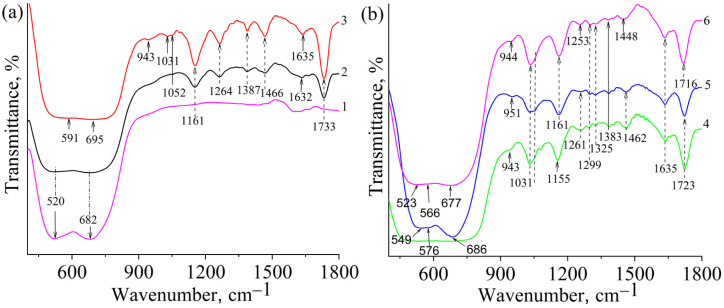
FTIR spectra of TiO_2_ (1), TiO_2_-P1 (2), TiO_2_-P2 (3), TiO_2_-P3 (4), TiO_2_-P10 (5) and TiO_2_-P14 (6).

**Fig. 5:**
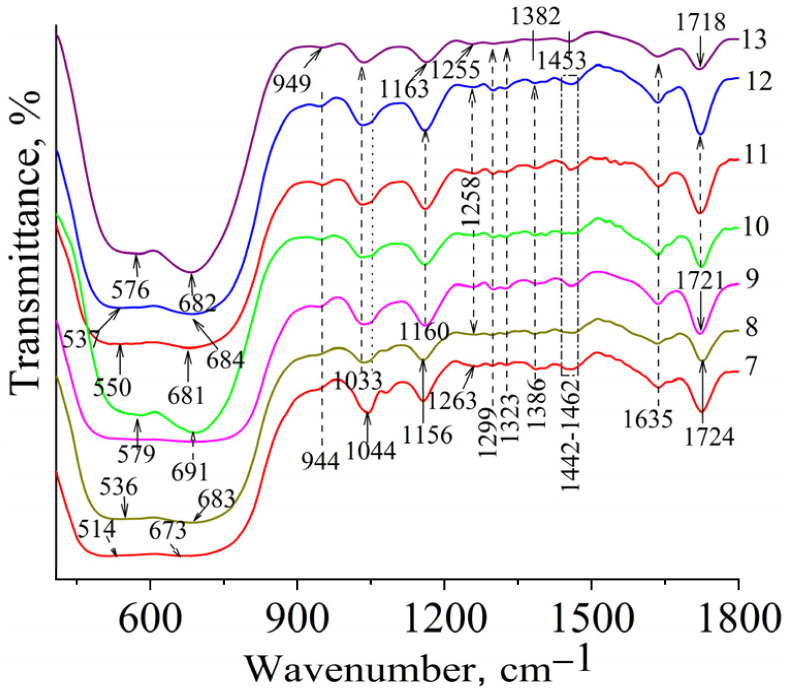
FTIR spectra of TiO_2_-P5Ag (7), TiO_2_-P8Ag (8), TiO_2_-P11Ag (9), TiO_2_-P10Ag (10), TiO_2_-P12Ag (11), TiO_2_-P13Ag (12), and TiO_2_-P15Ag (13).

In the FTIR spectra (Fig. 4 and 5) of the modified TiO_2_, the characteristic absorption bands are related to the stretching vibrations of the C–O bond of the glycoside ring (1031 cm^−1^) and the glycoside bridge (1155–1161 cm^−1^) of β-CD. Since the FTIR spectra of the sample based on maleoyl-β-CD (TiO_2_-P1) did not show the C–O valence band of the glycosidic ring of CD, we assumed that the copolymer formed mainly contained TMPTA units. The band at 1155–1161 cm^−1^ also belongs to the νC–O–C stretching vibrations of the ester groups of the methacrylate and copolymer. For example, in the spectrum of the sample prepared without TMTPA, the band at 1161 cm^−1^ has a slightly lower intensity than the other samples. In general, the intensity of this band increases slightly from the neat β-cyclodextrin to the copolymers. The νC=O stretching vibrations of the copolymer samples were observed at 1718–1730 cm^−1^. The characteristic valence bands of the C(=O)C–O methacrylate groups, which were used in particular to analyze the polymerization process, were observed at 1300, 1320 cm^−1^ and 1635 cm^−1^ of the C=CH_2_ fragment (*ν*_C=C_). The intensity of the band at 1251–1261 cm^−1^, which is attributed to the C(=O)C–O ester groups of the copolymer, increased compared with that of the initial monomer.^27^ The appearance of these bands in the spectra of the modified TiO_2_ confirms the formation of the polymer on its surface. The absorption bands of stretching bridging vibrations of Ti–O–Ti and stretching vibrations of Ti–O bonds are in the range of 450 to 800 cm^−1^ and appear as two bands at 520 cm^−1^ and 682 cm^−1^, respectively.^28–31^ In the spectra of modified TiO_2_, the band at 520 cm^−1^ was shifted (partially or completely) to higher frequencies (for the TiO_2_-P1 sample, it is not seen). Such a shift as observed in TiO_2_/Ag samples is generally due to the strengthening of Ti–O bonds, which, in our case, may be caused by an increase in crystallinity and compression of the crystal lattice. When titanium dioxide is doped with metal ions such as iron, cobalt, or silver, a shift of the Ti–O bond position toward the low-frequency region is often observed, indicating the weakening of this bond. This weakening of the oxide bonds is associated with the formation of oxygen vacancies in the TiO_2_ crystal lattice.^32–35^ Oxygen vacancies are formed during the formation of M–O bonds by substitution of Ti^4+^ with M^*n*+^. Such substitution creates a charge imbalance in the crystal lattice, and oxygen vacancies are formed to maintain charge neutrality. Instead, the formation of hydrogen bonds of the hydroxyl group on the surface of titanium dioxide with the groups of CD or copolymer, as well as the probable formation of Ti–O–C bonds, whose vibrations include the band at 943–952 cm^−1^,^36^ which appears in the spectra of samples synthesized based on β-CD methacrylates, would lead to a shift of the Ti–O bands to lower frequencies,^33,37^ which is associated with the weakening of the bonds due to changes in the electron density around oxygen atoms. The FTIR spectra of the silver-containing samples are shown in Fig. 5. A similar position of the bands was observed as for the starting copolymers.

**Fig. 6:**
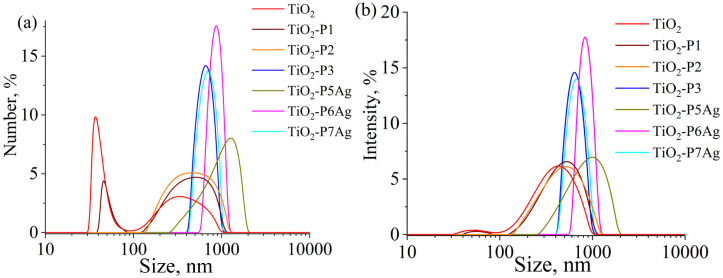
Particle size distributions by number (a) and intensity (b) of TiO_2_ and TiO_2_/CD copolymers.

Fig. 6 illustrates the particle size distribution of neat TiO_2_, TiO_2_/CDP, and TiO_2_/CDP/AgNPs obtained by UV reduction of Ag ions from its nitrate (P5Ag), methacrylate (P5Ag) and citrate (P6Ag).

The range of particle sizes (Table 2) for TiO_2_-P1 and P2 almost coincide with that of pure TiO_2_, but it is narrower for TiO_2_-P3, which was obtained from CD(Meth)_5_ and TMPTA and contains a larger number of CD moieties, as well as for the silver-containing samples TiO_2_-P6Ag and TiO_2_-P7Ag. The increase in particle size for these samples compared to pure TiO_2_ is most likely due to the formation of a 200–300 nm layer of cross-linked, branched polymer around the TiO_2_ particles rather than particle aggregation. For samples TiO_2_-P1 and P2, whose polymeric macromolecules contain practically no CD, the layer thickness can be estimated to be 50–70 nm, based on the increase in average particle size. It is also interesting to note that the sample prepared from Ag_Meth_ exhibited the lowest polydispersity index and the highest negative zeta potential value.

**Table 2:** Results of DLS analysis of the prepared composites

Sample	Mean particle size, nm	Hydrodynamic diameter, nm	Particle size range, nm	Polydispersity index	Mean zeta potential, mV
TiO_2_	55.4/421.4	379.3	34–953	0.23	−32.0
TiO_2_-P1	58.2/495.0	451.5	44–77/136–1034	0.25	−0.01
TiO_2_-P2	512.4	452.6	148–1216	0.23	−24.4
TiO_2_-P3	654.1	773.7	460–954	0.19	−25.6
TiO_2_-P5Ag	915.5	1077.9	283–1823	0.25	−25.2
TiO_2_-P6Ag	847.9	1072.2	636–1121	0.03	−35.5
TiO_2_-P7Ag	684.0	796.3	460–1034	0.22	−30.8

##### 3.2.2.1. XRD of TiO_2_/copolymers and TiO_2_/copolymers/AgNPs.

**Fig. 7:**
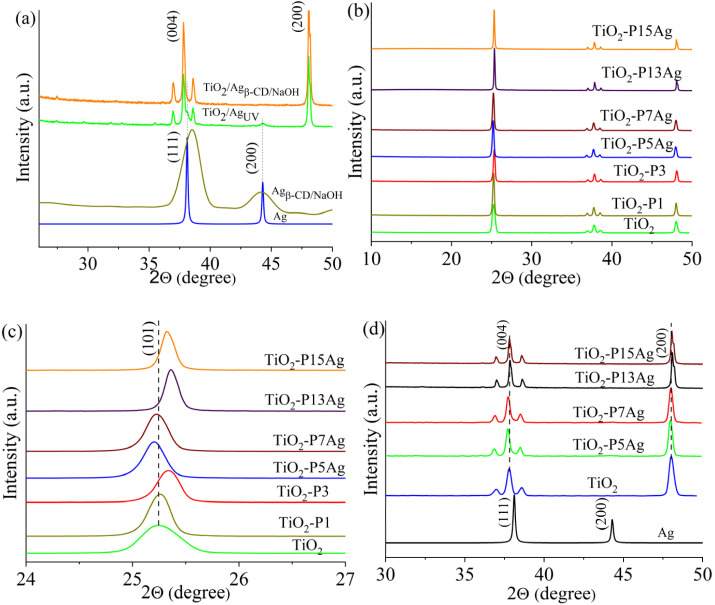
XRD patterns of TiO_2_, TiO_2_/AgNPs (a), TiO_2_/CDP (b), and TiO_2_/CDP/AgNPs (c and d – enlarged b).

The X-ray diffraction patterns of the TiO_2_, TiO_2_/CDP/AgNPs, TiO_2_/CDP, and TiO_2_/CDP/AgNP composites were studied (Fig. 7).

The XRD pattern of pristine TiO_2_ presents several sharp peaks at 2*θ* = 25.26, 37.80, 48.00 (Fig. 7b), which are assigned to the Miller indices (101), (004), (200) planes of the anatase phase according to JCPDS no. 21-1272.

For silver nanoparticles obtained in the presence of β-cyclodextrin from silver citrate (Fig. 7a), two broad maxima were observed at 38.5° and 44.3°. The crystallite size of Ag was 5.6 nm. For TiO_2_/Ag_uv_, the diffraction maxima were observed at 38.1°, 44.3°, 64.5, and 77.4 corresponding to the crystallographic planes of the silver face-centered cubic lattice with Miller indices (111), (200), (220), and (311), respectively (JCPDS card no. 04-0783).

The average crystallite size (*D*), interplanar spacing (*d*), and lattice parameters are listed in Table 3. The cyclodextrin-containing polymer AgNPs or their combination increased the crystallite size from 28.16 nm to 40.03–51.35 nm. However, their *d*-spacing and lattice parameters also changed. For example, for the sample containing practically no β-cyclodextrin moieties (TiO_2_-P1), the position of the reflexes and thus the lattice parameters do not change compared to the sample (TiO_2_-P4), while for the samples TiO_2_-P5Ag and TiO_2_-P7Ag the reflexes shift to smaller angles, indicating an increase in d-spacing and lattice parameters. This implies that silver atoms in these samples, in contrast to all other samples, may have replaced titanium or oxygen atoms. This assumption is consistent with the FTIR spectra data, in which changes in the Ti–O vibrational frequency range were observed, in particular the Ti–O–Ti band at 520 cm^−1^ toward lower frequencies, which is quite clearly seen in the example of the TiO_2_-P5Ag sample. For the other composites obtained by UV polymerization and reduction of silver ions, only a shoulder in the low-frequency region was found in the spectra, except for the sample obtained from silver methacrylate, for which, on the contrary, a clear shift toward 580 cm^−1^ occurred. That is, for samples TiO_2_-P5Ag and TiO_2_-P7Ag obtained by UV reduction of silver ions, the replacement of titanium atoms by silver atoms is more likely to occur, leading to an increase in the lattice parameters.

**Table 3:** Parameters of the structures of the studied composites

Sample	2*θ*, °	FWHM, °	*D*, nm	*d*, Å	Lattice parameters		
					*a*, Å	*c*, Å	*V*, Å^3^
TiO_2_	25.26	0.2891	28.16	3.521	3.783	9.627	137.77
TiO_2_/Ag_/nitr/CD/NaOH_	25.34	0.1622	50.21	3.510	3.779	9.476	135.32
TiO_2_/Ag_/meth/CD/NaOH_	25.33	0.1649	49.38	3.512	3.780	9.490	135.60
TiO_2_-P1	25.25	0.1933	42.11	3.522	3.785	9.623	137.86
TiO_2_-P3	25.34	0.2034	40.03	3.511	3.779	9.489	135.51
TiO_2_-P5Ag	25.20	0.1980	41.12	3.529	3.789	9.707	139.36
TiO_2_-P7Ag	25.23	0.1989	40.93	3.526	3.786	9.669	138.59
TiO_2_-P13Ag	25.37	0.1591	51.18	3.507	3.778	9.436	134.68
TiO_2_-P15Ag	25.33	0.1586	51.35	3.512	3.780	9.492	135.63

**Fig. 8:**
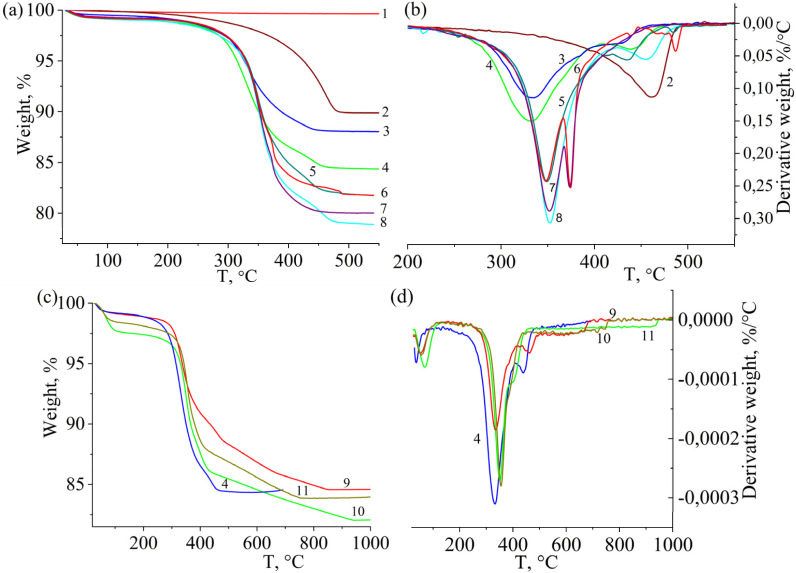
TGA (a and c) and DTG (b and d) curves of TiO_2_ (1), TiO_2_-P1 (2), TiO_2_-P8Ag (3), TiO_2_-P3 (4), TiO_2_-P13Ag (5), TiO_2_-P11Ag (6), TiO_2_-P10Ag (7), TiO_2_-P10 (8), TiO_2_-P3* (9), TiO_2_-P14* (10), and TiO_2_-P15-Ag* (11).

Fig. 8 shows the TGA curves of the pure and modified TiO_2_ composites. For all samples, the weight loss is insignificant in the first stage, which corresponds to moisture evaporation in the range of 0.2–1.1%, except for two samples, TiO_2_-P14 and TiO_2_-P15Ag, for which it was 2.4 and 1.6%. The degradation of the sample based on maleoyl-β-CD (TiO_2_-P1) proceeded in one stage with a maximum degradation rate at 459.5 °C. However, a rather slow degradation in the range of 280–390 °C without a distinguished stage can be attributed to the destruction of the CD moieties in the copolymer (≈2–3%). Instead, the destruction of the samples obtained from CD(Meth)_5_ (samples TiO_2_-P8Ag and TiO_2_-P3, curves 3 and 4) proceeded in two stages: the temperature range of 290–370°C corresponds to the destruction of β-CD and the methacrylate component, 370–460 °C is associated with the destruction of the aliphatic part of the copolymer, which for TMPTA links had a lower temperature (by ≈20–30°C) of the maximum thermal degradation rate than for sample TiO_2_-P1. According to the TGA data, the content of β-CD moieties in the copolymers was significantly lower during the polymerization of the maleoyl derivative than when methacrylate was used. For the pure β-CD, the maximum thermal degradation rate was 330 °C (*T*_onset_ = 316.8 °C).

When β-CD-(Meth)_5_ being applied, the cyclodextrin moieties in the samples degraded at almost the same temperature as the original β-CD-332–334 °C. Meanwhile, for the specimens prepared from 7-substituted acrylate (TiO_2_-P9) and methacrylate (TiO_2_-P10), the thermal stability of the macrocycle fragments increased by 16–22 °C.

The content of the organic component (copolymer) was determined based on the total weight loss and moisture, ranging from 9.9% for TiO_2_-P1 to 20.1% for TiO_2_-P10 (Table 4). The introduction of silver (samples TiO_2_-P8Ag, TiO_2_-P10Ag, TiO_2_-P11Ag, TiO_2_-P13Ag) slightly reduced the copolymer content, which may be due to a slight saponification of the ester groups since NaOH was utilized to reduce the silver ions. In addition, for the silver-containing composites, there is no distinguishable third stage in which the aliphatic component of the copolymer decomposes. The thermal analysis of TiO_2_-P14 and TiO_2_-P15Ag samples was performed at a lower heating rate (10 °C min^−1^) in a nitrogen atmosphere. For these composites, which were synthesized without TMPTA, the decomposition stage of the aliphatic component (methacrylate groups) was more pronounced and manifested over a wider temperature range (400–800 °C). The weight loss at this stage was consistent with the content of methacrylate groups in β-CD(Meth)_7_.

**Table 4:** Thermogravimetric analysis data

Composite	Weight loss 50–150 °C, %	*T*_onset_, °C	*T*_max_ (DTG), °C		Total weight loss, %
TiO_2_	0.16	—	—	—	0.4
TiO_2_-P1	0.21	398.6	—	459.5	10.1
TiO_2_-P3	0.81	289.8	331.9	437.9	15.6
TiO_2_-P3^a^	1.12	302.9	334.8	462.2	15.2
TiO_2_-P8Ag	0.60	297.3	334.4	—	12.0
TiO_2_-P9	0.29	284.7	346.6	442.4	11.6
TiO_2_-P10	0.98	325.0	352.4	455.1	21.1
TiO_2_-P10Ag	0.77	324.8	352.1	373.6	20.0
TiO_2_-P11Ag	0.85	315.0	347.3	374.2	18.2
TiO_2_-P13Ag	0.87	320.9	348.3	437.5	18.2
TiO_2_-P14^a^	2.40	319.5	350.1	—	17.9
TiO_2_-P15Ag^a^	1.60	324.4	354.4	—	16.1

a The samples were recorded under a nitrogen atmosphere.

#### 3.2.3. Textural parameters

According to the UPAC classification,^38^ the isotherms (Fig. S1†) can be classified as type IV isotherms, which are characterized by the presence of H3 hysteresis loops associated with capillary condensation in mesopores in the range of high relative pressure values. The specific surface area of the obtained samples increased in the following order: TiO_2_-P10 (6.24 m^2^ g^−1^) > TiO_2_-P11Ag (8.28 m^2^ g^−1^) > TiO_2_ (9.79 m^2^ g^−1^) > TiO_2_-P15Ag (10.14 m^2^ g^−1^). The average pore radius increased for modified TiO_2_ compared to pure TiO_2_ from 60.6 Å to 150.6 Å for TiO_2_-P11Ag. The TiO_2_-based samples exhibited a meso–macroporous structure with polymodal pores with an average pore size ranging from 2.4 to 60 nm in diameter. The pore volume distribution curves by size (DFT method) (Fig. S2†) are characterized by the presence of 4 peaks: 3 in the mesopore region (2.4–4.8 nm, 16.8–17.4 nm, 24.6–27.4 nm) and one in the macropore region (55.8–59.4 nm). Although there is no maximum in the micropore region on the pore distribution curve (DFT), the results of calculations using the DR, HK and SF methods, as well as the difference between the total pore volume and the mesopore volume (BJH/DH), confirm the presence of micropores in the sample. In our opinion, the correct value was obtained using the SF method because it was closest to the difference between *V*_total_ and *V*_meso_. The textural parameters of pure TiO_2_ and the synthesized composites are listed in Table 5.

**Table 5:** Textural parameters of the synthesized samples

Sample	Surface area, m^2^ g^−1^			Volume pore, cm^3^ g^−1^					Pore radius, Å				
	BET	BJH	DH	*V*_total_	*V*_meso_		*V*_micro_ SF	Micro-porous, %*V*_micro_/*V*_total_	*R*_av_	*R*			
					BJH	DH				*R*_micro_		DH	DFT
										DR	DA		
TiO_2_	9.79	6.69	6.68	0.0296	0.0275	0.0267	0.00294	9.9	60.6	7.9	8.0	15.0	18.3
TiO_2_-P10	6.24	2.86	2.90	0.0247	0.0230	0.0223	0.00181	7.3	79.3	11.3	8.9	48.2	12.1
TiO_2_-P11Ag	8.28	7.51	7.59	0.0623	0.0615	0.0597	0.00172	2.8	150.6	11.4	9.1	15.6	136.9
TiO_2_-P15Ag	10.14	7.53	7.61	0.0612	0.0593	0.0576	0.00264	4.3	120.6	12.3	9.2	14.8	18.3

The analysis of the pore distribution curves (Fig. S2†) shows that up to a pore size of 24–28 nm (radius 120–140 Å), the cumulative pore volume for all modified samples (TiO_2_-P10, TiO_2_-P11Ag, TiO_2_-P15Ag) is lower than that of the original TiO_2_. This indicates partial blocking of some micropores and mesopores (up to 28 nm). In the case of the TiO_2_-P10 sample containing only CD, the structure was probably compacted by filling the pores with polymer macromolecules. The observed increase in the cumulative volume of meso- and macropore regions for the samples with silver nanoparticles and CD polymers may be due to the formation of new pores in the polymer networks, but most likely it is caused by the unblocking of previously inaccessible pores in the range of 28–60 nm. This behavior could result from reduced particle aggregation or changes in surface wettability. These results indicate that the modification increases the accessible pore network, thereby improving the mass transport and photocatalytic properties.

### 3.3. Photocatalytic properties of the prepared composites

In this study, the photodegradation of MO was carried out at pH 2.5 because this is the most efficient method for initial TiO_2_ (ref. 39) degradation. In our opinion, the efficiency of MO decomposition at this pH is mainly due to the easier cleavage of the –N=N– azo group, which is predominantly in the protonated quinoid form –N–N= at pH 2.5, since the p*K*_a_ value of MO is 3.4.^40,41^ Although the adsorption of MO on the surface of the photocatalyst is also important, previous studies have shown that no adsorption occurred on the initial TiO_2_ at 2.5 for 30 min with stirring in the dark, and at pH 6, the adsorption was 1%. The effect of dark adsorption for TiO_2_-P10, TiO_2_-P11Ag and TiO_2_-P15Ag on the subsequent photodestruction of MO is shown in Fig. S3.† A slight decrease in the photodegradation of MO was observed for the TiO_2_-P10 and TiO_2_-P15Ag samples, for which the amount of adsorbed MO during 30 min was 4.5 and 6.4%, respectively. For the initial TiO_2_ and TiO_2_-P11Ag, adsorption as well as significant changes in the photodegradation process did not occur. Thus, the photodegradation deterioration was observed only in the case in which MO was previously adsorbed on the photocatalyst.

**Fig. 9:**
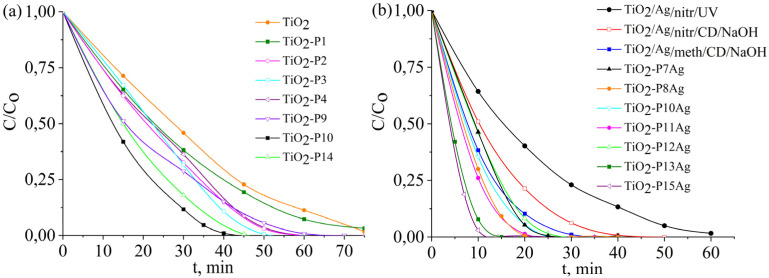
Photocatalytic degradation curves of methyl orange with TiO_2_, TiO_2_/CDP, TiO_2_/AgNPs, and TiO_2_/CDP/AgNPs (conditions: *C*_0_ (MO) = 25 mg L^−1^, *V* = 80 ml, and pH 2.5).

**Fig. 10:**
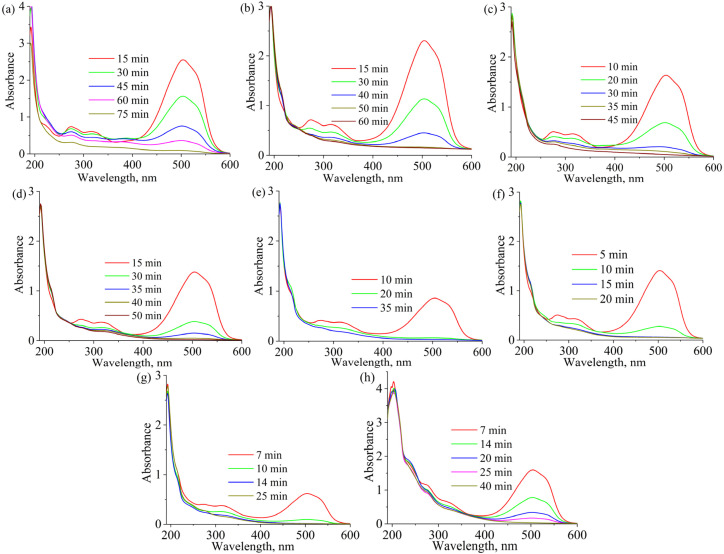
UV-vis spectra of MO solutions after irradiation using TiO_2_ (a), TiO_2_-P3 (b), TiO_2_/Ag_/Citr/CD_ (c), TiO_2_-P10 (d), TiO_2_-P11Ag (e), TiO_2_-P13Ag (f), TiO_2_-P15Ag 1st cycle (g), and TiO_2_-P15Ag 5th cycle (h).

Fig. 9a and b illustrate the photodegradation curves of methyl orange at pH 2.5 in artesian water, and Fig. 10a–h the corresponding UV-vis spectra during irradiation for copolymers on TiO_2_, the best results were obtained with samples based on β-CD(Meth)_7_ (TiO_2_-P10), due to the presence of a greater number of macrocycles on the TiO_2_ surface than in composites prepared with other β-CD monomers. A comparison of the polymerization methods showed that the samples obtained by UV polymerization exhibited slightly higher photocatalytic activity (TiO_2_-P3 and TiO_2_-P4, respectively).

Two kinetic models were used to study the mechanism of MO photodegradation in the presence of prepared photocatalysts: pseudo-zero (1) and pseudo-first order (2) kinetic models, which are described by the following equations:1
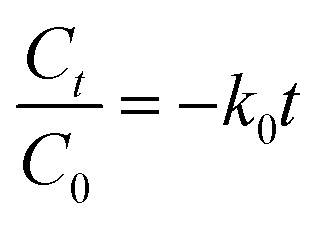
2
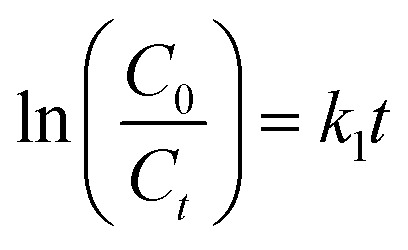
where *C*_0_ i *C*_*t*_ are the initial and at the definite time *t* (min) concentration of methyl orange, mg L; *k*_0_ is the rate constant of the pseudo-zero kinetic model, mg l^−1^ min^−1^; *k*_1_ is a rate constant of the pseudo-first kinetic model, min^−1^. In the pseudo-zero-order kinetic model,^50^ the photodegradation rate is controlled only by the irradiation time, whereas in the pseudo-first-order kinetic model, the chemosorption is the limiting step. The *R*^2^ value for each equation is higher than 0.95, indicating that the experimental data are satisfactory fitted to the pseudo-first and pseudo-second order models, with the TiO_2_/Ag samples showing a better fit to the pseudo-first order model, while the TiO_2_/CD polymer and TiO_2_/CD polymer/Ag samples mostly show a better fit to the pseudo-second order model (Table 6).

**Table 6:** Kinetic parameters of MO degradation by the prepared photocatalyst

Photocatalyst	Kinetic model					
	Pseudo-first order			Pseudo-zero order		
	Equation	*K*_1_ (min^−1^)	*R* ^2^	Equation	*k*_0_ mg l^−1^ min^−1^	*R* ^2^
TiO_2_	*y* = 0.0367*x* − 0.1461	0.0367	0.9762	*y* = −0.0151*x* + 0.9548	0.0151	0.9801
TiO_2_/Ag_Nitr_/CD	*y* = 0.092*x* − 0.1318	0.092	0.9814	*y* = −0.0311*x* + 0.913	0.0311	0.9439
TiO_2_/Ag_Cit_/CD	*y* = 0.0912*x* − 0.1137	0.0912	0.9856	*y* = −0.031*x* + 0.9066	0.031	0.9366
TiO_2_/Ag_Meth_/CD	*y* = 0.1136*x* − 0.0587	0.1136	0.992	*y* = −0.0448*x* + 0.9438	0.0448	0.955
TiO_2_-P1	*y* = 0.0467*x* − 0.2333	0.0467	0.979	*y* = −0.0129*x* + 0.8722	0.0129	0.9235
TiO_2_-P2	*y* = 0.0462*x* − 0.1086	0.0462	0.9638	*y* = −0.0212*x* + 0.9755	0.0212	0.9941
TiO_2_-P3	*y* = 0.0539*x* − 0.1982	0.0539	0.9203	*y* = −0.0225*x* + 1.0017	0.0225	0.9996
TiO_2_-P4	*y* = 0.0527*x* − 0.2047	0.0527	0.9206	*y* = −0.0201*x* + 0.9714	0.0201	0.994
TiO_2_-P5Ag	*y* = 0.1195*x* − 0.1577	0.1195	0.9504	*y* = −0.0454*x* + 0.98	0.0454	0.9942
TiO_2_-P6Ag	*y* = 0.1138*x* − 0.3137	0.1138	0.8734	*y* = −0.0386*x* + 1.0069	0.0386	0.9989
TiO_2_-P7Ag	*y* = 0.1467*x* − 0.233	0.1467	0.9297	*y* = −0.0473*x* + 0.9791	0.0473	0.9942
TiO_2_-P8Ag	*y* = 0.1534*x* − 0.0826	0.1534	0.9664	*y* = −0.0619*x* + 0.9797	0.0619	0.9872
TiO_2_-P9	*y* = 0.0548*x* − 0.1413	0.0548	0.9614	*y* = −0.0184*x* + 0.8974	0.0184	0.9432
TiO_2_-P10	*y* = 0.0843*x* − 0.1634	0.0843	0.9618	*y* = −0.027*x* + 0.935	0.027	0.965
TiO_2_-P10Ag	*y* = 0.1449*x* − 0.1374	0.1449	0.9737	*y* = −0.0472*x* + 0.9424	0.0472	0.9573
TiO_2_-P11Ag	*y* = 0.2132*x* − 0.2628	0.2132	0.9564	*y* = −0.0493*x* + 0.9179	0.0493	0.9232
TiO_2_-P12Ag	*y* = 0.1246*x* − 0.1568	0.1246	0.9547	*y* = −0.0459*x* + 0.973	0.0459	0.9897
TiO_2_-P13Ag	*y* = 0.2551*x* − 0.1364	0.2551	0.9668	*y* = −0.0922*x* + 0.9605	0.0922	0.9785
TiO_2_-P14	*y* = 0.0571*x* − 0.0542	0.0571	0.9881	*y* = −0.0273*x* + 0.9698	0.0273	0.984
TiO_2_-P15Ag	1st cycle *y* = 0.3314*x* − 0.15	0.3314	0.9361	*y* = −0.1004*x* + 0.9748	0.1004	0.9795
	5th cycle *y* = 0.1167*x* − 0.0699	0.1167	0.9953	*y* = −0.0368*x* + 0.8613	0.0368	0.902

To determine the effect of the β-CD macrocycle on the photocatalytic properties of silver-containing samples, the study was first carried out on samples containing only silver nanoparticles reduced from different silver salts in the presence and absence of β-CD. The presence of β-CD moieties during the reduction of silver ions on the TiO_2_ surface contributed to the formation of a material with better photocatalytic properties for all the studied salts and, to a greater extent, in the case of silver nitrate. In general, more efficient photodestruction was observed for the TiO_2_/AgNPs_/meth/CD_ sample obtained from methacrylate in the presence of β-CD (Fig. 9b). Fig. 9b shows the results of the photodegradation studies of MO for samples containing both the copolymer and silver NPs. For the samples based on β-CD(Meth)_7_ and silver methacrylate (Fig. 9b), the destruction was the most effective, with the discoloration of MO occurring within 12 min, which is also evident from the corresponding UV spectra taken during the destruction process (Fig. 10e and f). A similar pattern was observed for TiO_2_-P15Ag prepared from silver methacrylate without TMPTA. The decomposition of the MO degradation by-products was also more efficient for these samples, as evidenced by the absence of a noticeable shoulder at 276 and 320 nm, as for the original copolymers. The degradation of products with maximum absorption at these wavelengths is also clearly visible from the UV-vis spectra at the 5th irradiation cycle (4 cycles of 25 min each) (Fig. 10h). The products formed because of the photodegradation of methyl orange significantly inhibit this process in the following cycles. By the 5th cycle, the decomposition time of the main dye molecule was twice as long. If the solution is separated from the photocatalyst or the degradation products are effectively decomposed, for instance by adding hydrogen peroxide,^20,42^ potassium peroxymonosulfate^43^ or peroxydisulfate,^44^ the photocatalytic efficiency will not decrease. Table 7 summarizes the results of the efficiency of silver-containing TiO_2_-based photocatalysts for the photodegradation of MO and other pollutants.

**Table 7:** Comparison of the photoactivity of TiO_2_-based nanocomposites

Photocatalyst	Experimental conditions	Light source	Photodegradation efficiency	Ref.
Ag/MoO_3_/TiO_2_	*C* (MO) = 10 mg L^−1^	UV lamp 100 W	95.6%	42
	*C* (Catalyst) = 0.96 g L^−1^		99% (H_2_O_2_)	
	*V* = 125 ml		*t* = 330 min	
	pH = 3.0			
TiO_2_–Ag(1%)-potassium persulfate 1.87 mM	*C* (MO) = 100 mg L^−1^	UV lamp	95,83%	43
	*C* (Catalyst) = 0.66 g L^−1^		*t* = 20 min	
	*V* = 100 ml			
	pH = 2, 6			
Ag@SiO_2_/TiO_2_ peroxydisulfate 0.45 mM	*C* (MO) = 5 mg L^−1^	Xenon lamp	99%	44
	*C* (Catalyst) = 0.6 g L^−1^		*t* = 120 min	
	*V* = 100 ml			
	pH = 7.25			
TiO_2_/40AgI	*C* (MO) = 5 mg L^−1^	10 W LED (*λ* = 430 nm)	100%	45
	*C* (Catalyst) = 1.5 g L^−1^		*t* = 20 min	
	*V* = 20 ml			
TiO_2_/Ag (7.2%) composite microspheres	*C* (MO) = 10 mg L^−1^	15 W, *λ* = 365 nm, 395 nm	96.84% and 98.65%	46
	*C* (Catalyst) = 1.5 g L^−1^		*t* = 120 min	
	*V* = 40 ml			
TiO_2_–AgI–cotton	*C* (MO) = 5 mg L^−1^	1000 W Xe lamp (*λ* > 400 nm)	56%	47
	*C* (Catalyst) = 1.25 g L^−1^		*t* = 120 min	
	*V* = 50 ml			
Ag–AgI(4%)–TiO_2_/carbon nanofibers	*C* (MO) = 10 mg L^−1^	300 W Xe arc lamp (*λ* ≧ 400 nm	97%	48
	*C* (Catalyst) = 1 g L^−1^		*t* = 180 min	
	*V* = 100 ml			
AgI–TiO_2_/PAN	*C* (MO) = 10 mg L^−1^	300 W Xe arc lamp	87.8%	49
	*C* (Catalyst) = 2 g L^−1^		*t* = 270 min	
	*V* = 100 ml			
g-C_3_N_4_/Ag-TiO_2_	*C* (MO) = 10 mg L^−1^	300 W Xe lamp with a UV cut filter and an 18 W UV-A lamp	74.92% and 92.82%	50
	*C* (Catalyst) = 0.42 g L^−1^		*t* = 60 min	
	*V* = 120 ml			
TiO_2_/0.3Ag//porous polymer	*C* (MO) = 10 mg L^−1^	Xenon lamp	81.4%	51
	*C* (Catalyst) 0.5 g L^−1^		84.8%	
	*V* = 100 ml		*t* = 180 min	
TiO_2_/0.5Ag/biochar	*C* (MO) = 20 mg L^−1^	500 W mercury-vapor lamp 360 nm	97.48%	52
	*C* (Catalyst) 0.25 g L^−1^		*t* = 60 min	
	*V* = 40 ml			
TiO_2_/Ag (4%)	*C* (MO) = 16 mg L^−1^	8 W Hg vapor lamp (365 nm)	98.9% (UV)	53
	*C* (Catalyst) = 1 g L^−1^		*t* = 60 min, 99.3% solar irradiation	
	*V* = 500 ml		*t* = 80 min	
TiO_2_/β-cyclodextrin polymer/Ag (TiO_2_-P15Ag)	*C* (MO) = 25 mg L^−1^	UV lamp 24 W 365/254 nm	97% *t* = 10 min (1st cycle)	This work
	*C* (Catalyst) = 0.5 g L^−1^		94.6% *t* = 25 min (5th cycle)	
	*V* = 80 ml, pH = 2.5			
β-CDP/TiO_2_	*C* (TC) = 50 mg L^−1^	Solar light irradiation	96%	7
	*C* (Catalyst) = 0,5 g L^−1^		*t* = 90 min	
CMCD-Fe_3_O_4_@TiO_2_	*C* (BPA, DBP) = 20 mg L^−1^	Mercury vapor lamp (400 W)	100%	54
	*C* (Catalyst) 0,5 g L^−1^ pH 7–10, V = 15 ml		*t* = 105 min	
TiO_2_/CMCD	*C* (BPA) = 20 mg L^−1^	UV lamp 24 W	100%	55
	*C* (Catalyst) 0,25 g L^−1^		*t* = 120 min	
	*V* = 40 ml			
TiO_2_–La 0.05%–CMCD	*C* (MB) = 10 mg L^−1^	80 W high pressure mercury lamp, 254 nm	95%	12
	*C* (Catalyst) 1 g L^−1^ pH 6, V = 15 ml		*t* = 15 min	
β-CD/PNC/TiO_2_	*C* (MB) = 5 mg L^−1^	500 W xenon lamp with 350 nm cut-off filter	83.7%	9
	*C* (Catalyst) = 0.5 g L^−1^		*t* = 180 min	
	*V* = 100 ml			
β-CD/TiO_2_	*C* (MO) = 32 mg L^−1^	250 W high-pressure mercury lamp	100%	56
	*C* (Catalyst) = 1 g L^−1^		*t* = 20 min	
	*V* = 100 ml			
Ce/TiO_2_/CD polymer	*C* (RhB) = 5 mg L^−1^	500 W halogen lamp	80%	57
	*V* = 50 ml		*t* = 60 min	
Fe–N–TiO_2_/β-CD	*C* (AO7) = 20 mg L^−1^	10 W 400–800 nm (daylight white light, UV-LED at 365 nm)	MB: 94% (UV) *t* = 60 min	58
	*C* (MB) = 7 mg L^−1^		50% (visible light) *t* = 180 min	
	*C* (Catalyst) = 1 g L^−1^		AO7: 99% (UV)	
	*V* = 100 ml		*t* = 60 min	

## 4. Conclusions

Hybrid composites TiO_2_/Ag and TiO_2_/β-cyclodextrin polymer/Ag were developed using β-CD methacrylate or maleate with different degrees of substitution, trimethylolpropane trimethacrylate as a crosslinking agent, and various silver salts (nitrate, methacrylate, citrate). Studies have shown that the use of TiO_2_/CD polymer composites doped with silver nanoparticles significantly enhances the efficiency of the photocatalytic decomposition of methyl orange dye. The photodegradation kinetics of methyl orange fit well to a pseudo-zero-order model, and the rate constant (*k*_0_) increases by up to 6.5 times compared with the initial TiO_2_. This improvement is attributed to the ability of cyclodextrin's cavity to stabilize silver nanoparticles and enhance pollutant adsorption on the catalyst surface. Using XRD and FTIR spectroscopy, it was found that the introduction of silver nanoparticles led to changes in the crystal structure of TiO_2_ and improved separation of electron-hole pairs, as confirmed by a shift in the valence vibrational frequencies of Ti–O bonds. This phenomenon also influences the photodegradation efficiency of the hybrid composites mentioned above, suggesting the possibility of optimizing the catalyst composition.

## Data availability

The data that support the findings of this study are available from the corresponding author upon reasonable request.

## Author contributions

Serhii Kobylinskyi: investigation, writing – original draft, conceptualization, and formal analysis; Sergii Sinelnikov: investigation and resources; Larysa Kobrina: investigation and visualization; Yuliia Bardadym: investigation and visualization; Sergii Riabov: conceptualization, writing – review and editing, project administration, and supervision. All the authors have read and agreed to the published version of the manuscript.

## Conflicts of interest

There are no conflicts to declare.

## Supplementary Material

RA-015-D4RA08901D-s001
