# Supplementary material for: Silver-containing composites based on copolymers of β-cyclodextrin and TiO2 for enhanced photocatalytic degradation of methyl orange in environmental water
Source: RSC Adv. 2025 May 30;15(23):17955–71. doi: 10.1039/d4ra08901d (PMC12123402; doi:10.1039/d4ra08901d)
Supplement: RA-015-D4RA08901D-s001 [file RA-015-D4RA08901D-s001.pdf]

Supplementary Information (SI) for RSC Advances.
This journal is © The Royal Society of Chemistry 2025

Supplementary information

Silver-containing composites based on copolymers of β-cyclodextrin and TiO_2 for enhanced photocatalytic degradation of methyl orange in environmental water

Serhii Kobylinskyi, Sergii Sinelnikov, Larysa Kobrina, Yuliia Bardadym, Sergii Riabov*

Institute of Macromolecular Chemistry, National Academy of Sciences of Ukraine,
48, Kharkivske shose, Kyiv 02155, Ukraine, e-mail: sergii.riabov@gmail.com*

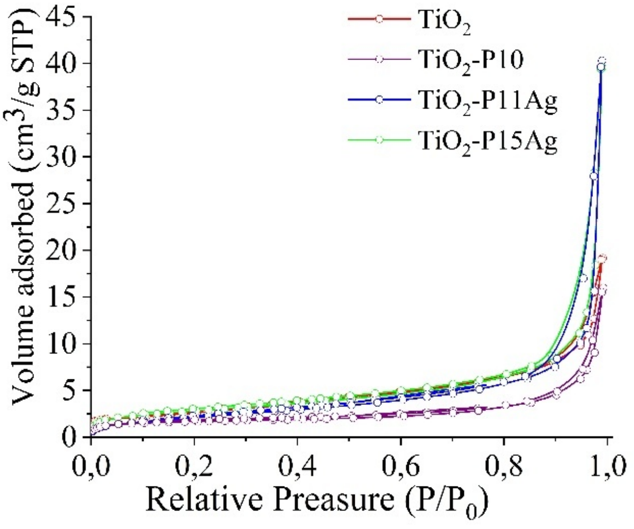

Fig. S1 Nitrogen adsorption/desorption isotherms of TiO_2, TiO_2-P10, TiO_2-P11Ag, TiO_2-P15Ag

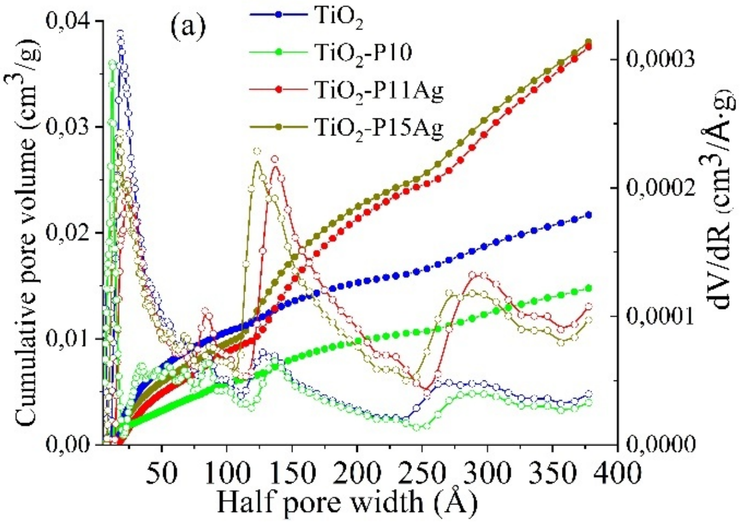

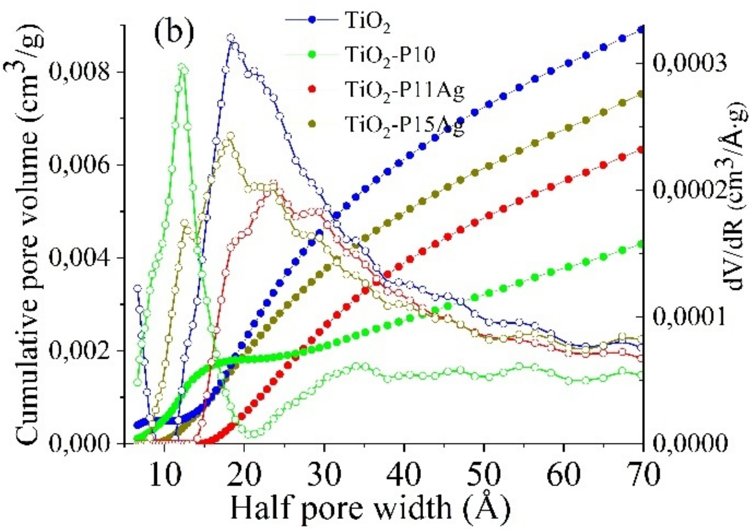

Fig. S2 DFT cumulative pore volume and pore size distribution of TiO_2, TiO_2-P10, TiO_2-P11Ag, TiO_2-P15Ag

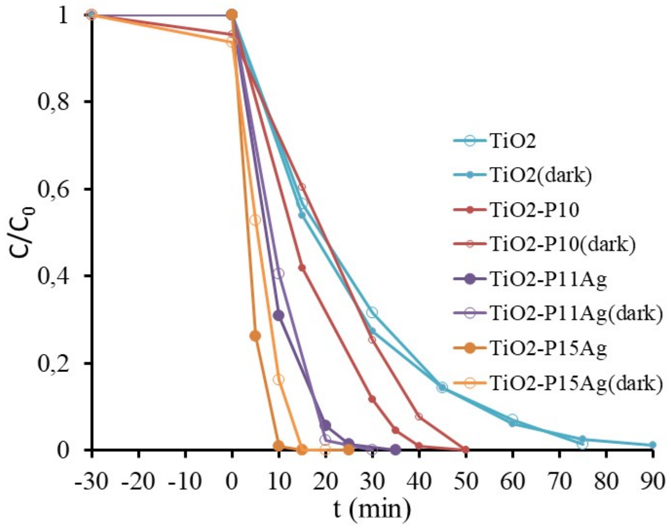

Fig. S3 Photocatalytic degradation curves of methyl orange
